# The Value of Blood Examinations in Appendicitis

**Published:** 1903-10-10

**Authors:** 


### THE VALUE OF BLOOD EXAMINATIONS IN APPENDICITIS.

Dr. M. Cazin and Dr. E. Gros[1] have made an extended series of observations on the condition of the blood in appendicitis with a view to determining, if possible, the precise value of leucocytosis as a symptom in this affection. For practical purposes they consider leucocytosis to be present whenever the number of white corpuscles in the blood exceeds 10,000 per cubic millimetre. They have found that leucocytosis is usually present at the very beginning of an attack of appendicitis, even in the absence of suppuration. In slight cases the increase in the number of white corpuscles is not pronounced, the number varying between 10,000 and 15,000, but sometimes reaching 20,000 by the second or third day, even in the absence of localised suppuration. This increase, however, is very evanescent, and rapidly disappears. These observations agree, as Dr. Cazin and Dr. Gros point out, with the experimental results obtained by Schultz, who showed that, in the dog, simple irritation of the peritoneum by an aseptic incision through the lima alba followed by immediate closure, brought about occasionally very marked leucocytosis. Wassermann, too, noticed that laparotomy for the removal of the appendix during a period of quiescence was usually followed by leucocytosis; the proportion of the white corpuscles although quite normal before the operation may rise afterwards to 14,000 or even 20,800, ultimately falling to normal, without there having been the slightest suspicion of sepsis. It is therefore demonstrated that simple irritation of the peritoneum, apart altogether from the presence of micro organisms, determines leucocytosis. Therefore every attack of appendicitis, accompanied by the least peritoneal reaction, is followed by an increase in the number of leucocytes, except in certain hypertoxic forms where the individual for some reason or other fails to show a reaction.

At the onset of the attack no difference is noticeable, in regard to the leucocytosis, between the cases which end in resolution and those which terminate in suppuration. Mere enumeration of the white corpuscles cannot therefore afford any assistance at the beginning of an attack of appendicitis towards a diagnosis of the course that the illness is likely to follow; and it is only by methodical enumeration repeated morning and evening that it is possible to distinguish cases which tend to resolution from those in which an abscess is forming. In mild cases ending in resolution the leucocytosis is as a rule never severe. Out of 17 of such instances in which the enumeration was carried out systematically, in only six did the number exceed 15,000, and the highest count was 22,900. For practical purposes it may be said that, if the number of leucocytes undergoes only a slight increase, in the majority of instances the course of the illness will be mild and comparatively short, and that, if the number during several consecutive days remain comparatively low, resolution may be expected even when the temperature fails to point to such a prognosis. It is in the diagnosis of suppuration that the blood counts have their greatest value. A persistent high leucocytosis constituting a reliable and invaluable sign of the presence of an abscess, even where the temperature is normal or but slightly raised. All the published observations show that there is no definite relationship between temperature and leucocytosis, and when the two point to different conclusions it is to the latter that the greater importance must be attached, although this sign is not invariably present in cases of suppuration. There are two types of bad cases in which the number of white corpuscles may not be markedly increased. In the one, which Dr. Cazin and Dr. Gros call the hypertoxic form, the defensive reactions of the individual affected are wanting, there is no tendency to localisation of the septic process, and the case is rapidly fatal. In the other there is an abscess present, but it is shut in by a very thick fibrous wall, and it is on account of this, perhaps, that the leucocytosis does not occur. There is another practical and important fact which is shown by enumeration of the leucocytes in respect of the post-operative prognosis. After an appendix abscess has been opened there is generally a rapid disappearance of the leucocytosis, the number of white corpuscles reaching the normal often within the next 24 or 48 hours. If, therefore, this return to the normal does not occur, some complication must be suspected; perhaps a separate collection of pus which was overlooked at the operation. From these observations it will be seen that repeated blood counts may be of very great practical value, where a single examination of the blood might fail to give much useful information, although one blood count may be of assistance in excluding other possible conditions which are not characterised by leucocytosis, while if at the single blood examination a high degree of leucocytosis be found it constitutes an indication for operative interference.

[1] Medical Press, Sept. 30.

